# Cephalometric evaluation of the effects of the Twin Block appliance in subjects with Class II, Division 1 malocclusion amongst different cervical vertebral maturation stages

**DOI:** 10.1590/2177-6709.21.3.073-084.oar

**Published:** 2016

**Authors:** Aisha Khoja, Mubassar Fida, Attiya Shaikh

**Affiliations:** 1 Resident in Orthodontics, The Aga Khan University Hospital, Section of Dentistry, Department of Surgery, Karachi, Pakistan.; 2 Associate Professor, Program Director Orthodontics Residency Program, The Aga Khan University Hospital, Section of Dentistry, Department of Surgery, Karachi, Pakistan.; 3 Assistant Professor, Program Coordinator Orthodontics Residency Program, Section of Dentistry, Department of Surgery, The Aga Khan University Hospital, Karachi, Pakistan.

**Keywords:** Twin Block, Class II, Division 1 malocclusion, Cervical vertebral maturation

## Abstract

**Objectives:**

To evaluate the cephalometric changes in skeletal, dentoalveolar and soft tissue variables induced by Clark's Twin Block (CTB) in Class II, Division 1 malocclusion patients and to compare these changes in different cervical vertebral maturation stages.

**Methods:**

Pre- and post-treatment/observation lateral cephalograms of 53 Class II, Division 1 malocclusion patients and 60 controls were compared to evaluate skeletal, dentoalveolar and soft tissue changes. Skeletal maturity was assessed according to cervical vertebral maturation stages. Pre- and post-treatment/observation mean changes and differences (T_2_-T_1_) were compared by means of Wilcoxon sign rank and Mann-Whitney U-tests, respectively. Intergroup comparisons between different cervical stages were performed by means of Kruskal-Wallis test and Mann-Whitney U-test (*p* ≤ 0.05) .

**Results:**

When compared with controls, there was a significant reduction in ANB angle (*p* < 0.001), which was due to a change in SNB angle in CS-2 and CS-3 (*p* < 0.001), and in SNA (*p* < 0.001) and SNB (*p* = 0.016) angles in the CS-4 group. There was significant increase in the GoGn-SN angle in CS-2 (*p* = 0.007) and CS-4 (*p* = 0.024), and increase in Co-Gn and Go-Gn amongst all cervical stages (*p* < 0.05). There was significant decrease in U1-SN and increase in IMPA amongst all cervical stages (*p* < 0.05). There was significant retraction of the upper lip in CS-3 (*p* = 0.001), protrusion of the lower lip in CS-2 (*p* = 0.005), increase in nasolabial angle in CS-4 (*p* = 0.006) and Z-angle in CS-3 (*p* = 0.016), reduction in H-angle in CS-2 (*p* = 0.013) and CS-3 (*p* = 0.002) groups. When pre- and post-treatment mean differences were compared between different cervical stages, significant differences were found for SNA, SNB and UI-SN angles and overjet. .

**Conclusions:**

The Twin-Block along with the normal craniofacial growth improves facial esthetics in Class II, Division 1 malocclusion by changes in underlying skeletal and dentoalveolar structures. The favorable mandibular growth occurs during any of the cervical vertebral maturation stages, with more pronounced effect during CS-3 stage.

## RESUMO

**Objetivo:**

avaliar as alterações em variáveis cefalométricas esqueléticas, dentoalveolares e do perfil tegumentar, induzidas pelo aparelho *Twin Block* de Clark (TBC), em pacientes com má oclusão de Classe II, divisão 1, e comparar as alterações nos diferentes estágios de maturação das vértebras cervicais.

**Métodos:**

telerradiografias laterais pré- e pós-tratamento de 53 pacientes com má oclusão de Classe II, divisão 1, foram comparadas às telerradiografias de 60 pacientes controle, para avaliar as alterações esqueléticas, dentoalveolares e no perfil tegumentar. A maturação esquelética foi avaliada de acordo com os estágios de maturação das vértebras cervicais. As alterações médias e as diferenças entre o pré- e o pós-tratamento (T_2_ - T_1_) foram comparadas por meio do teste de postos sinalizados de Wilcoxon e do teste U de Mann-Whitney, respectivamente. Foram realizadas comparações intergrupos para os diferentes estágios de maturação das vértebras cervicais, por meio do teste de Kruskal-Wallis e teste U de Mann-Whitney (*p* ≤ 0,05).

**Resultados:**

em comparação aos pacientes do grupo controle, constatou-se que houve uma redução significativa do ângulo ANB (*p* < 0,001), em virtude de alterações no ângulo SNB nos estágios CS2 e CS3 (*p* < 0,001), e nos ângulos SNA (*p* < 0,001) e SNB (*p* = 0,016) no estágio CS4. Houve um aumento significativo do ângulo GoGn-SN nos estágios CS2 (*p* = 0,007) e CS4 (*p* = 0,024), e um aumento em Co-Gn e Go-Gn em todos os estágios de maturação das vértebras cervicais (*p* < 0,05). Houve redução significativa em U1-SN e um aumento do IMPA em todos os estágios de maturação das vértebras cervicais (*p* < 0,05). Houve retração significativa do lábio superior em CS3 (*p* = 0,001), protrusão do lábio inferior em CS2 (*p* = 0,005), aumento do ângulo nasolabial em CS4 (*p* = 0,006) e do ângulo Z em CS3 (*p* = 0,016), além de redução do ângulo H em CS2 (*p* = 0,013) e CS3 (*p* = 0,002). Quando as diferenças médias entre pré- e pós-tratamento foram comparadas entre os diferentes estágios de maturação das vértebras cervicais, foram identificadas diferenças significativas para os ângulos SNA, SNB e UI-SN, assim como para o *overjet*.

**Conclusões:**

o uso do aparelho *Twin-Block*, associado ao crescimento craniofacial normal, melhora a estética facial em pacientes com má oclusão de Classe II, divisão 1, por meio de alterações nas estruturas esqueléticas e dentoalveolares subjacentes. Esse crescimento mandibular mais favorável pode ocorrer durante qualquer um dos estágios de maturação das vértebras cervicais, com um efeito mais acentuado durante o estágio CS3.

## INTRODUCTION

Physical attractiveness plays a vital role in social interaction and in dealing with people in society.^1^ The face is the first structure to be noticed and people with well-proportioned and attractive faces are perceived as being more outgoing, friendly, socially competent, optimistic, intelligent, and confident.^2^

Subjects with Class II, Division 1 malocclusion typically present with an increased overjet, lower lip trapped behind maxillary incisors and an unfavorable facial profile, which may predispose children towards a negative feeling of self-image and self-esteem.^3^ ^-^ ^6^ The goal of orthodontic treatment for these patients is to achieve a harmonious relationship of dentoskeletal subunits along with an esthetically pleasing facial profile.^3^ ^,5^

Class II malocclusion is commonly observed by orthodontists in daily practice.^7^ In a local study conducted by Gul-e-Erum and Fida,^8^ 70.5% of patients had Angle Class II, and amongst them 64.7% had Class II, Division 1 malocclusion. On a global scale, an approximate estimation shows over 20% prevalence of Class II malocclusion in North America, Europe and North Africa.^9^

Various treatment modalities can be instituted to treat these patients, amongst which functional appliance has been found to be a suitable treatment option in growing individuals.^10^ ^,^ ^11^These appliances work by changing the activity of the various muscle groups that influence function and position of the mandible.^12^ Altering sagittal and vertical mandibular position generates pressure due to stretching of muscles and surrounding soft tissues. The resultant force is transmitted to the underlying dental and skeletal tissues and brings about orthodontic and orthopedic changes.^13^ Twin Block is the most preferred type of functional appliance in the United Kingdom.^3^ ^,^ ^10^It was first introduced by Clark, in 1982,^14^ and has been increasingly popular because of its uncomplicated design and ease of use.^10^ It consists of two separate upper and lower acrylic units which position the mandible forward through interlocking occlusal bite blocks.^10^ ^,^ ^13^The independent units facilitate speech and mastication and are proved to be associated with good patient compliance.^12^ ^,^ ^13^

A multitude of evidence-based studies have described the role of the Twin Block appliance on skeletal, dental and soft tissue structures.^3^ ^,^ ^10^ ^,^ ^11^ ^,^ ^15^ ^,^ ^16^Some studies^3^ ^,^ ^16^ ^,^ ^17^suggest that functional appliance can increase mandibular growth, provided it is used in the growing age, whereas others^18^ ^,^ ^19^did not find any real change in the length of the mandible. Nevertheless, dental changes have been observed by most researchers.^3^ ^,^ ^10^ ^,^ ^16^ ^,^ ^17^ ^,^ ^20^ To the best of our knowledge, no prospective clinical trials have been conducted in Pakistan to investigate the clinical effects of functional appliances in Class II, Division 1 malocclusion patients. However, there was a review article by Sukhia^21^ on the jasper jumper appliance, its usage, effects and modifications. Therefore, the primary aim of this research is to assess the mean changes in skeletal, dentoalveolar and soft tissue variables on lateral cephalogram at a one-year interval in growing individuals with Class II, Division 1 malocclusion following Twin Block appliance therapy. Early intervention in these patients promotes the growth of the mandible in a favorable manner, thereby resulting in a pleasing facial profile. This will provide children with psychosocial advantage; in addition, the subsequent need for orthodontic tooth extractions and orthognathic surgery will be minimized. Moreover, these children may also exhibit less signs and symptoms of temporomandibular joint dysfunction by repositioning the condyles downward and forward.^22^

The effectiveness of functional appliances at inducing skeletal changes largely depends on the growth rate of the mandible. The stages of cervical vertebral maturation are directly related to mandibular growth changes that occur during puberty. The stages include observations during the accelerated growth phase (CS-1 and CS-2) and observations during the decelerated phase (stages CS4, CS-5 and CS-6).^23^ The peak in pubertal growth occurs on average between vertebral stages 3 and 4. Evidence has been gathered from the literature, suggesting that the greatest effect of functional appliance is produced when it is used during the peak in mandibular growth.^23^ ^,^ ^24^ However, there is variable response to treatment in different subjects at different cervical vertebral maturation stages. Hence, it is important to evaluate the cervical stage of an individual before intervening with the functional appliance. Therefore, the secondary goal of this study is to evaluate the effects of the Twin Block appliance on skeletal, dental and soft tissues in Class II, Division 1 patients treated at different cervical vertebral maturation stages (CS-2, CS-3, and CS-4).

## MATERIAL AND METHODS

Sample size was calculated keeping α = 0.05, power of study (β) as 81% and by using the findings of a study conducted by Toth and McNamara.^25^ They reported pre- and post-treatment mean differences for the variable Co-Gn (mandibular unit length) in the Twin Block group (5.7 ± 2.4 mm) and in the control group (2.7 ± 1.5 mm). Power analysis showed a minimum sample of 51 subjects. After considering the rate of lost to follow-up as well as non compliant patients, we included 65 consecutive patients.

Ethical approval to conduct this study was obtained from the Ethical Review Committee of Aga Khan University Hospital (AKUH), Karachi Pakistan (2910-Sur-ERC-14). After taking informed consents from the parents and assents from the children, a total of 65 consecutive children were recruited for this study. All of them met the following inclusion criteria:

1) Skeletal Class II relationship measured on cephalometric radiograph (ANB > 5°).

2) Mandibular retrognathism measured on cephalometric radiograph (SNB < 78°).

3) Class II incisor, canine and molar relationships.

4) Overjet ≥ 6 mm. 

5) Patients of growing age (9-16 years)who were in CS-2, CS-3 and CS-4 of cervical vertebral maturation stages, according to Baccetti et al.^23^

The exclusion criteria of this study were subjects with any craniofacial anomaly or syndrome, noncompliant or uncooperative patients who failed to wear the appliance for more than 12 hours/day, and subjects with history of orthodontic treatment. The compliance to wear the appliance for a minimum of 12 hours/day was monitored by asking the patient and his/her parents on every visit and later confirming it with the help of an overjet change. If there was no improvement in overjet for two consecutive months, it clearly indicated failure to wear the appliance. 

A total of 12 patients were excluded from the total sample. Seven patients failed to wear the appliance for more than 12 hours/day, three patients did not follow up after appliance delivery and an additional two presented with frequent appliance breakage. Hence, we ended up with a final sample of 53 patients among which 25 were males and 28 were females. 

The control group consisted of 60 subjects (30 males, 30 females) selected from the Bolton Brush growth study and had no history of orthodontic treatment. These subjects were matched in skeletal age (according to the cervical vertebral maturation stages), sex, dental malocclusion, overjet and ANB angle with the experimental subjects. The mean observation period for the control group was taken at one-year interval to match with the post-treatment readings of the study group. 

For the experimental group, data were obtained from the lateral cephalograms taken at the beginning (T_1_) and at the end (T_2_) of full time appliance wear of patients presented at AKUH dental clinics. The Twin Block appliance was manufactured according to the original design described by Clark, with the modification of mandibular incisor capping. Construction bite was recorded with the mandible postured forward into an edge-to-edge incisal relationship with 2-3 mm of interincisal clearance and 5-6 mm of bite opening in the first premolar region. Patients with pretreatment overjet greater than 7 mm had stepwise mandibular advancement performed. Initially, the bite was registered in the range of 4-6 mm, followed by reactivation of an appliance in an end-to-end incisal position after a few months. Reactivation of appliance was carried out by adding cold cure acrylic on the anterior incline of upper Twin Block halfway through treatment.^25^ ^,^ ^26^All patients were instructed to wear the appliance full time for a period of 8-12 months, except during brushing and meal times. In addition, all appliances incorporated a midline expansion screw which was activated 0.25 mm every alternate day by means of a slow expansion technique.

**Figure 1:**
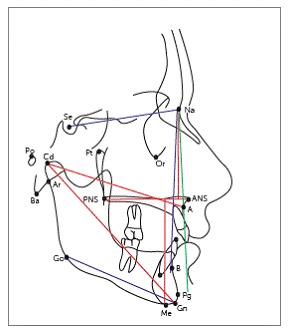
Skeletal variables.^37^

**Figure 2:**
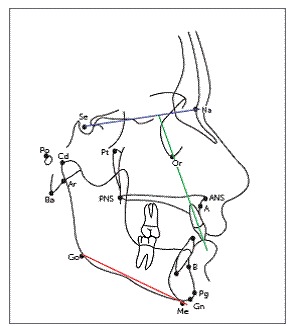
Dentoalveolar variables.^37^

**Figure 3:**
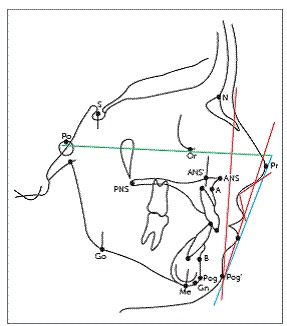
Soft tissue variables.^37^

Pre- and post-treatment cephalograms were manually traced on acetate paper over an illuminator by the main investigator, according to the conventional method. Several landmarks were marked, over which various linear and angular measurements were taken to evaluate skeletal, dental and soft tissue changes (Figs 1-3). Overjet was measured clinically on each visit, as the distance from the labial surface of mandibular central incisor to the labial surface of the most prominent maxillary incisor, with the help of an overjet scale. Skeletal maturity stages were assessed on lateral cephalogram by observing the morphological and dimensional changes of the bodies of second through sixth cervical vertebrae, according to the evaluation method by Baccetti et al.^23^

In order to ensure a high degree of precision, the pre- and post-treatment lateral cephalograms of subjects were routinely taken with the sagittal plane at right angle to the path of x-ray beams, the head in an erect position, Frankfort horizontal plane being parallel to the ground, teeth in centric occlusion and lips lightly closed in a relaxed position. These radiographs were recorded with rigid head fixation and a 165-cm film-to-tube distance by means of Orthoralix^TM^ 9200 (Kavo Gendex, Milan, Italy).

## STATISTICAL ANALYSIS

Statistical analyses were performed with Statistical Package for the Social Sciences (SPSS) for Windows (version 19.0 Chicago Inc. USA). Descriptive statistics (mean and SD) were computed for all quantitative variables. Shapiro-Wilk test was used to check for normality of data, showing a non-normal distribution for most variables. Wilcoxon signed rank test was applied to compare changes in skeletal, dentoalveolar and soft tissue cephalometric variables from T_1_ to T_2_ in the treatment and control groups. The mean differences were then compared by means of Mann-Whitney U-test between treatment and control groups. 

The sample was further stratified into three cervical vertebral maturation groups (CS-2, CS-3 and CS-4). Pre- and post-treatment (T_2_-T_1_) mean differences for each variable were calculated amongst these groups and were later compared with untreated controls which were also selected on the basis of cervical vertebral maturation stages using the same nonparametric tests. 

To assess the effects of the Twin Block appliance, used at different cervical vertebral maturation stages, pre- and post-treatment mean differences (T_2_-T_1_) were compared for skeletal, dental and soft tissue variables by means of the Kruskal-Wallis test. Intergroup comparisons (between CS-2 and CS-3, CS-2 and CS-4, CS-3 and CS-4) were carried out for the cephalometric variables by means of Mann-Whitney U-test. Level of significance was set at *p* ≤ 0.05.

## ERROR ANALYSIS

To detect any error in locating different landmarks on lateral cephalogram and in measuring pre- and post-treatment skeletal, dental and soft tissue changes, replicated measurements separated by four weeks in 20 randomly selected pre- and post-treatment cephalograms were performed by the main investigator (intraexaminer error). The intraclass correlation coefficient denoted that repeated measurements were strongly correlated with correlation values greater than 0.90.

## RESULTS

A total of 53 pre- and post-treatment cephalograms of Class II, Division 1 malocclusion patients (28 males, 25 females) and 60 pre- and post observational cephalograms of controls (24 males, 36 females) were compared to investigate the overall changes in skeletal, dentoalveolar and soft tissue variables. The mean age of males and females in the treatment group was 11.4 ± 1.71 and 11.8 ± 1.62 years, respectively. The mean age for males and females in controls were 11.1 ± 1.68 and 11.2 ± 1.86 years, respectively. 

### Pre- and post-treatment/observation mean changes in treatment and control groups in the total sample

Initial compatibility between treatment and control groups was examined by comparison of cephalometric variables at T_1_, as shown in Table 1. 

**Table 1:** Comparison between treatment and control groups at T_1_.

Variables	Treatment group (T_1_) Mean ± SD	Control group (T_1_) Mean ± SD	*p* value
SNA	81.1 ± 3.88	81.8 ± 2.07	0.351
SNB	73.8 ± 3.41	74.7 ± 2.15	0.182
ANB	7.31 ± 1.99	7.12 ± 2.19	0.316
GoGn-SN	32.9 ± 4.63	33.9 ± 4.81	0.198
Co-A	86.7 ± 4.81	87.9 ± 5.82	0.134
Co-Gn	106.3 ± 6.92	106.3 ± 7.29	0.968
Go-Gn	67.4 ± 4.24	67.4 ± 6.70	0.266
UI-SN	109.7 ± 9.82	108.1 ± 6.45	0.109
IMPA	101.4 ± 7.16	100.2 ± 5.70	0.580
OJ (overjet)	8.37 ± 1.97	7.87 ± 2.98	0.146
UL-Eline	-0.22 ± 1.67	-0.27 ± 2.85	0.764
LL-Eline	0.83 ± 2.74	-0.01 ± 3.72	0.221
N-L angle	102.8 ± 13.3	105.6 ± 7.47	0.552
Z-angle	60.5 ± 5.68	62.3 ± 5.10	0.352
H-angle	23.7 ± 4.51	23.0 ± 3.31	0.804

Mann-Whitney U-test.

* *p* < 0.05.

Pre- and post-treatment/observation means and standard deviations of the cephalometric skeletal, dentoalveolar and soft tissue variables in treatment and control groups are presented in Table 2.

**Table 2:** Pre- and post-treatment/observation changes in skeletal, dental and soft tissue variables.

Variables	Treatment group (n = 53)			Control group (n = 60)		
	T_1_Mean ± SD	T_2_Mean ± SD	*p* value	T_1_Mean ± SD	T_2_Mean ± SD	*p* value
Skeletal variables						
SNA	81.1 ± 3.88	80.9 ± 4.00	0.180	81.8 ± 2.07	81.9 ± 1.80	0.555
SNB	73.8 ± 3.41	75.5 ± 3.54	< 0.001**	74.7 ± 2.15	74.8 ± 2.19	0.072
ANB	7.31 ± 1.99	5.37 ± 1.99	< 0.001**	7.12 ± 2.19	6.98 ± 2.23	0.455
GoGn-SN	32.9 ± 4.63	33.5 ± 5.38	0.189	33.9 ± 4.81	33.8 ± 4.62	0.615
Co-A	86.7 ± 4.81	87.8 ± 5.06	< 0.001**	87.9 ± 5.82	88.4 ± 5.70	0.057
Co-Gn	106.3 ±6.92	110.9± 7.89	< 0.001**	106.4 ±7.29	107.7 ± 7.06	< 0.001**
Go-Gn	67.4 ± 4.24	70.8 ± 4.22	< 0.001**	67.4 ± 6.70	68.4 ± 8.63	0.206
Dentoalveolar variables						
UI-SN	109.8 ± 9.82	105.1 ± 8.60	< 0.001**	108.1 ± 6.45	109.2 ± 9.82	0.002*
IMPA	101.4 ± 7.16	105.8 ± 6.31	< 0.001**	100.2 ± 5.70	101.3 ± 5.60	0.124
Overjet	8.37 ± 1.97	1.86 ± 1.41	< 0.001**	7.87 ± 2.98	7.56 ± 3.43	0.067
Soft tissue variables						
UL-E-line	-0.23 ± 1.67	-1.03 ± 2.55	0.014*	-0.27 ± 2.85	-1.29 ± 1.79	0.433
LL-E-line	0.83 ± 2.74	1.21 ± 2.58	0.095	-0.00 ± 3.72	-0.56 ± 3.34	0.194
N-L angle	102.8 ± 13.3	106.4 ± 11.6	0.022*	105.6 ± 7.47	101.8 ± 10.4	0.084
Z-angle	60.5 ± 5.68	62.8 ± 7.45	< 0.001**	62.3 ± 5.10	61.3 ± 5.59	0.585
H-angle	23.7 ± 4.51	20.2 ± 3.20	< 0.001**	23.0 ± 3.31	22.8 ± 3.11	0.620

Wilcoxon signed rank test.

* *p* < 0.05; ** *p* < 0.001.

From these measurements, the mean difference (post-treatment/observation - pretreatment) was then calculated for each variable in treatment and control groups. The change in the study group was then compared to the natural growth change in the control group by means of Mann-Whitney U-test, as shown in Table 3. Treatment effect was calculated by subtracting natural craniofacial growth from the treatment change. The results showed a significant increase in SNB angle (*p* < 0.001), decrease in ANB angle (*p* < 0.001), and increase in vertical jaw relationship (*p* = 0.029), increase in mandibular unit length and body (*p* < 0.001). Amongst the dentoalveolar structures, there was significant reduction in overjet (*p* < 0.001) and maxillary incisor inclination (*p* < 0.001), whereas mandibular incisor incisors inclination increased (*p* < 0.001). There was statistically significant retraction of upper lip with respect to the E-line (*p* = 0.015), increase in N-L (*p* = 0.001) and Z-angle (*p* < 0.021), and a decrease in the H-angle (*p* < 0.001). 

**Table 3:** Mean change in cephalometric variables between treatment and control group (T_2_-T_1_).

Variables	Treatment group (n = 53)	Control group (n = 60)	Treatment effect	*p* value
	Mean ± SD	Mean ± SD	(Treatment - Control group)	
SNA	-0.19 ± 1.10	0.04 ± 1.01	-0.23	0.168
SNB	1.73 ± 1.22	0.17 ± 1.03	1.56	< 0.001**
ANB	-1.96 ± 1.16	-0.14 ± 1.21	-1.82	< 0.001**
GoGn-SN	0.60 ± 2.45	-0.19 ± 1.09	0.79	0.029*
Co-A	1.13 ± 2.06	0.52 ± 2.08	0.61	0.068
Co-Gn	4.58 ± 2.97	1.31 ± 2.28	3.27	< 0.001**
Go-Gn	3.45 ± 2.24	0.52 ± 2.06	2.93	< 0.001**
UI-SN	-4.66 ± 5.44	1.12 ± 4.19	-5.78	< 0.001**
IMPA	4.30 ± 3.91	1.05 ± 3.45	3.25	< 0.001**
OJ (overjet)	-6.50 ± 2.46	-0.30 ± 1.25	-6.20	< 0.001**
UL-E-line	-0.81 ± 2.41	-0.62 ± 3.47	-0.19	0.015*
LL-E-line	0.37 ± 1.57	-0.55 ± 4.24	0.92	0.082
N-L angle	3.64 ± 9.83	-3.72 ± 14.17	7.36	0.001*
Z-angle	2.30 ± 3.89	-1.07 ± 8.12	3.37	0.021*
H-angle	-3.56 ± 4.86	-0.20 ± 2.72	-3.36	< 0.001**

Mann-Whitney U-test.

* *p* < 0.05; **p < 0.001.

### Comparison of pre- and post-treatment/observation mean changes in treatment and control groups at different cervical stages

The sample was further stratified into three groups, on the basis of cervical vertebral maturation stages, into CS-2, CS-3 and CS-4 in both treatment and control groups. Pre and post-treatment/observation mean difference (post-treatment/observation - pretreatment) for each variable was then compared between treatment and control groups by means of Mann-Whitney U-test, so as to identify the actual treatment effect, as shown in Table 4. The results showed an overjet correction of 5.0, 7.4 and 6.0 mm in CS-2, CS-3 and CS-4 groups, respectively. When compared with untreated subjects at similar cervical stages, there was statistically significant reduction in ANB angle amongst the three cervical stage groups (*p* < 0.001). However, this reduction was primarily due to change in SNB angle in CS-2 (*p* < 0.001) and CS-3 (*p* < 0.001) groups, and in both SNA (*p* < 0.001) and SNB (*p* = 0.016) angles in the CS-4 group. In vertical dimension, there was a significant increase in the mandibular plane angle in relation to the S-N plane in CS-2 (*p* = 0.007) and CS-4 (*p* = 0.024) groups. The change in mandibular unit length and body was significant in CS-2 (*p* < 0.001), CS-3 (*p* < 0.001, *p* = 0.001) and CS-4 (*p* = 0.027, *p* = 0.004) groups. Amongst the dentoalveolar variables, there was statistically significant reduction in maxillary incisor inclination and increase in mandibular incisor inclination in CS-2 (*p* < 0.001, *p* = 0.002), CS-3 (*p* = 0.013, *p* = 0.005) and CS-4 (*p* < 0.001, *p* = 0.005) groups when compared with their controls. Upper lip retraction was significant in CS-3 (*p* = 0.001), whereas lower lip became more projected in CS-2 (*p* = 0.005). The nasolabial angle increased significantly in CS-4 (*p* = 0.006) and Z-angle in CS-3 (*p* = 0.016); whereas reduction in H-angle was significant in CS-2 (*p* = 0.013) and CS-3 (*p* = 0.002) stages when compared with their control groups, respectively.

**Table 4:** Pre- and post-treatment/observation mean changes (T_2_-T_1_) between treatment and controls amongst different cervical stages.

Variables	CS-2			CS-3			CS-4		
	Mean ± SD			Mean ± SD			Mean ± SD		
	TG	CG	*p* value	TG	CG	*p* value	TG	CG	*p* value
	n = 18	n = 20		n = 22	n = 20		n = 13	n = 20	
SNA	-0.47 ± 0.81	-0.88 ± 0.66	0.194	0.32 ± 1.28	0.58 ± 0.75	0.682	-0.69 ±0.75	0.41 ± 0.90	< 0.001**
SNB	1.44 ± 1.04	-0.39 ±1.07	< 0.001**	2.32 ± 1.28	0.73 ± 0.63	< 0.001**	1.15 ± 0.98	0.18 ± 1.03	0.016*
ANB	-1.92 ± 1.03	-0.48 ± 1.28	0.001*	-2.00 ± 1.27	-0.15 ± 1.27	< 0.001**	-1.84 ±1.21	0.23 ± 1.00	< 0.001**
GoGn-SN	0.27 ± 2.02	-0.52 ± 1.21	0.007*	0.14 ± 2.55	-0.28 ± 0.97	0.629	1.84 ± 2.57	0.23 ± 1.00	0.024*
Co-A	0.94 ± 2.71	0.07 ± 1.57	0.194	1.50 ± 1.33	0.25 ± 2.70	0.290	0.77 ± 2.12	1.24 ± 1.70	0.956
Co-Gn	3.72 ± 1.74	1.03 ± 1.93	< 0.001**	5.54 ± 3.26	1.24 ± 2.92	< 0.001**	4.15 ± 3.53	1.65 ± 1.93	0.027*
Go-Gn	3.38 ± 1.68	0.10 ± 1.57	< 0.001**	3.59 ± 2.59	0.29 ± 2.73	0.001*	3.31 ± 2.46	1.17 ± 1.62	0.004*
UI-SN	-6.72 ± 6.22	1.05 ± 6.29	< 0.001**	-1.68 ± 4.30	1.30 ± 2.90	0.013*	-6.77 ±3.51	1.00 ± 2.55	< 0.001**
IMPA	4.55 ± 4.09	1.88 ± 2.05	0.002*	3.00 ± 3.10	0.61 ± 2.38	0.005*	6.15 ± 4.35	0.67 ± 5.10	0.005*
OJ	-5.59 ± 2.96	-0.55 ± 1.41	< 0.001**	-7.25 ± 2.20	0.14 ± 1.01	< 0.001**	-6.51 ±1.73	-0.52 ± 1.24	< 0.001**
UL-E-line	0.05 ± 2.76	0.04 ± 1.54	0.597	-1.18 ± 1.10	-1.26 ± 5.64	0.001*	-1.38 ±3.25	-0.65 ± 1.54	0.096
LL-E-line	1.05 ± 1.39	-0.76 ± 2.04	0.005*	0.04 ± 1.49	-0.25 ± 7.03	0.630	0.00 ± 1.73	-0.65 ± 1.41	0.426
N-L angle	4.33 ± 9.14	-2.55 ± 15.3	0.208	2.31 ± 10.53	-1.80 ± 13.3	0.164	5.53 ± 10.9	-6.80 ± 14.4	0.006*
Z-angle	1.33 ± 3.94	-0.10 ± 5.24	0.407	2.30 ± 3.89	-1.06 ± 8.12	0.016*	2.46 ± 4.96	-0.90 ± 10.2	0.781
H-angle	-3.50 ± 3.89	-0.70 ± 3.22	0.013*	-4.54 ± 6.35	-0.40 ± 2.25	0.002*	-2.00 ±2.41	-0.30 ± 2.61	0.162

Mann-Whitney U-test. *p < 0.05; **p < 0.001. CG= Control group; TG= Treatment group.

### Comparison of pre- and post-treatment mean differences (T **_2_** -T **_1_** ) in the treatment group at different cervical stages

To assess variability in the effect of the Twin Block appliance in Class II subjects treated at different cervical stages, pre- and post-treatment mean differences (T_2_-T_1_) were compared for cephalometric skeletal, dental and soft tissue variables between CS-2, CS-3 and CS-4 stages of the treatment group. There was statistically significant difference in the variables SNA (*p* = 0.010), SNB (*p* = 0.020), UI-SN (*p* = 0.003) and overjet (*p* = 0.035) between the three cervical vertebral maturation groups. Intergroup comparisons were further performed by means of multiple comparison tests to evaluate pre- and post-treatment (T_2_-T_1_) changes at different cervical stages, as shown in Table 5.

**Table 5:** Pre- and post-treatment changes (T_2_-T_1_) in cephalometric variables at different cervical stages.

Cephalometric variables				*p* value^#^	Multiple comparisons for the cephalometric variables		
	CS-2 (n = 18) Mean ± SD	CS-3 (n = 22) Mean ± SD	CS-4 (n = 12) Mean ± SD		CS-2/CS-3 p ^†^	CS-2/CS-4 p^†^	CS-3/CS-4 p^†^
SNA	-0.47 ± 0.81	0.32 ± 1.28	-0.69 ± 1.73	0.010*	0.016*	0.435	0.011*
SNB	1.44 ± 1.04	2.32 ± 1.28	1.15 ± 0.98	0.020*	0.037*	0.540	0.015*
ANB	-2.00 ± 1.02	-2.00 ± 1.27	-1.84 ± 1.21	0.910	0.735	0.885	0.699
GoGn-SN	0.27 ± 2.02	0.14 ± 2.55	1.84 ± 2.57	0.096	0.339	0.266	0.026*
Co-A	0.94 ± 2.71	1.50 ± 1.33	0.77 ± 2.12	0.617	0.363	0.792	0.490
Co-Gn	3.72 ± 1.74	5.54 ± 3.26	4.15 ± 3.53	0.171	0.064	0.840	0.236
Go-Gn	3.38 ± 1.68	3.59 ± 2.59	3.31 ± 2.46	0.900	0.890	0.625	0.769
UI-SN	-7.16 ± 6.67	-1.68 ± 4.30	-6.76 ± 3.51	0.003*	0.010*	0.904	0.002*
IMPA	4.55 ± 4.09	3.00 ± 3.10	6.15 ± 4.35	0.065	0.056	0.387	0.055*
OJ	-5.59 ± 2.96	-7.25 ± 2.20	-6.52 ± 1.73	0.035*	0.018*	0.088	0.264
UL-E-line	0.05 ± 2.76	-1.18 ± 1.10	-1.38 ± 3.25	0.244	0.475	0.183	0.128
LL-E-line	1.05 ± 1.39	0.04 ± 1.49	0.00 ± 1.73	0.057	0.032*	0.057	0.696
N-L angle	4.88 ± 9.79	2.32 ± 10.53	4.92 ± 10.05	0.431	0.261	0.936	0.295
Z-angle	1.33 ± 3.94	3.00 ± 3.10	2.46 ± 4.96	0.480	0.227	0.559	0.619
H-angle	-3.50 ± 3.89	-4.54 ± 6.35	-2.00 ± 2.41	0.441	0.701	0.162	0.437

# = Kruskal-Wallis test; ^†^ = Mann-Whitney U-test.

* *p* ≤ 0.05

CS-2 = Cervical stage 2; CS-3 = Cervical stage 3; CS-4 = Cervical stage 4.

## DISCUSSION

Class II malocclusion can manifest in various combinations of skeletal and dental disharmony that affect the overlying soft tissue facial profile. However, the majority of patients have anteroposterior deficiency of the mandible.^27^ Gillmore^28^ reported a retropositioned, small mandible in patients with Class II, Division 1 malocclusion. Therefore, an ideal treatment plan for these patients is primarily directed towards functional appliance.

In this study, changes in skeletal, dentoalveolar and soft tissue variables were measured on lateral cephalograms following Twin Block appliance therapy. In order to assess the influence of normal growth that would have occurred without the appliance in place, it is important to have a control group.^29^ Various authors have used different control groups, such as Class II, Division 1 malocclusion patients,^5^ ^,^ ^19^ Class I patients who did not require treatment,^30^ ^,^ ^31^ patients whose pretreatment records have been done, but they refused to continue treatment,^3^ and published normative data using Bolton and Michigan growth standards.^25^ ^,^ ^29^ An ideal control group should be similar in terms of malocclusion, age, sex, race, skeletal maturity and an equal observation period to that of the treatment group. Therefore, in order to match the control group with the study group as precise as possible, published normative growth data were used and retrieved from the Bolton Brush study.

In order to determine the sole effects of the Twin Block appliance, multi-banded fixed orthodontic appliances were not placed during the active and supporting phase of treatment. The results of this study showed that the Twin Block appliance has a short term effect in treating Class II, Division 1 malocclusion by a combination of skeletal (instant forward shift of the mandible, increase in mandibular unit length and body, gonial angle changes) and dental effects (maxillary incisor retroclincation and by loss of anterior anchorage of mandibular incisors). 

### Effects on the maxilla

O'Brien et al^16^ found minimal restraining effect on maxillary growth with the Twin Block appliance, which constituted 13% of overall skeletal changes. Similarly, Illing et al^20^ also demonstrated a small mean reduction in SNA angle. Due to the stretch of the muscles and surrounding soft tissues of the facial skeleton, the forwardly placed mandible tends to return to its original position. This creates a reciprocal restraining effect on the maxilla, which is called headgear effect.^13^ ^,^ ^27^ However, several other studies did not find any significant orthopedic effect exerted on the maxilla with this appliance.^27^ ^,^ ^28^ The results obtained in the present study are in concordance with their study results, with no statistically significant reduction in SNA angle. In addition, change in maxillary unit length (Co-A) was also insignificant. Nevertheless, on stratification of sample into different cervical stages, significant reduction in SNA angle was found in the CS-4 stage when compared with controls. Toth and McNamara^25^ reported that the studies supporting maxillary growth restriction have included extraoral force along with functional appliance. In addition, construction bite, when registered in a single step, produces headgear effects due to stretch of the retractor muscles. 

### Effects on the mandible

The effect of functional appliance on mandibular growth is controversial. Several studies have suggested that functional appliance can increase the SNB angle by anterior relocation of point B and pogonion.^10^ ^,^ ^20^Baysal and Uysal^3^ found a significant increase in SNB angle after treatment with the Twin Block appliance. Illing et al^20^ found an increase in mandibular unit length measured from point condylion and articulare to gnathion. Toth and McNamara^25^ found an increase in mandibular unit length (Co-Gn) of 3.0 mm during a 16-month period when compared with controls. Our results are similar to the aforementioned studies, with significant increase in SNB angle by 1.56˚ and mandibular unit length of 3.27 mm over a 12-month period. Growth stimulation by the Twin Block appliance produced a greater change over a short treatment duration, which is of benefit to the patients.^27^ However, it was not possible to identify whether the increase in point condylion to gnathion was due to true increase in mandibular length or merely a repositioning of the mandible. In addition, no actual measurements of mandibular fossa adaptation or relocation were made in this study. Therefore, it is recommended that further studies be conducted to assess the long term effects of the Twin Block appliance on mandibular growth increments as well as to see the role of mandibular fossa adaptation and possible relocation with the functional appliance.

When skeletal changes were compared among subjects at different cervical vertebral maturation stages, in a study conducted by Baccetti et al,^32^ greater changes were observed in the late treated groups (CS-3 and CS-4), as compared to the early treated groups (CS-1 and CS-2). The greater therapeutic effectiveness of functional appliance occurs during the peak in the pubertal growth spurt of an individual, which coincides with the maximum growth rate of the mandible.^33^ Similarly, Malmgren et al^34^ found greater skeletal effects of Bass appliance in boys treated during the peak period than those treated during the prepeak period. In our study, we also observed greater mandibular skeletal changes in CS-3 and CS-4 groups, as compared to the CS-2 group. However, this increase was statistically insignificant. 

### Maxillomandibular changes

In light of evidence, it was found that the reduction in ANB angle following Twin Block appliance therapy may occur by decrease in SNA and increase in SNB or both. Toth and McNamara^25^ found reduction in ANB angle by 1.8˚ in patients treated with the Twin Block appliance. Likewise, Illing et al^20^ found statistically significant reduction in ANB angle, as compared to controls. Our results are similar to the above findings, with mean reduction in ANB angle by 1.82˚ in the total sample. This reduction in ANB angle was primarily due to an increase in SNB angle in CS-2 and CS-3 groups; whereas, in CS-4, it occurred due to a combination of decrease in SNA angle and increase in SNB angle.

### Vertical relationship of the jaws

There is large variability in treatment response, with a few studies showing an increase in total anterior facial height and maxillary-mandibular plane angle (MMPA); whereas other studies demonstrated a small mean reduction in mmPA angle.^16^ ^,^ ^17^ ^,^ ^25^ ^,^ ^35^ The possible reason for this decrease in mmPA is inhibition of molar eruption by increasing the height of the posterior bite blocks or by rotation of maxillary plane.^20^ In this study, a significant increase in vertical jaw relationship (GoGn-SN) was found, as compared to the controls following Twin Block appliance therapy. However, on stratification of sample into different cervical stages, this increase was significant at CS-2 and CS-4 stages, as compared to controls. Since the authors of this study did not consider the vertical dimensions of subjects prior to their inclusion, this may have affected treatment results. Therefore, it is advisable that subjects in future studies be selected with regard to their facial heights and vertical pattern of growth. 

### Dentoalveolar changes

Illing et al^20^ found a mean reduction in the inclination of maxillary incisors, which was more pronounced in the Twin Block group (-9.1 ± 6.2˚) when compared to Bass and bionator. This effect is greater by incorporation of labial bow into an appliance. O'Brien et al^16^ showed that maxillary incisor retraction contributed significantly to overjet reduction and, therefore, Class II malocclusion is mainly corrected by dentoalveolar movements rather than mandibular growth. In our study, significant retroclination of maxillary incisors was found following Twin Block appliance therapy amongst all cervical stages. However, this reduction in maxillary incisor inclination was greater in CS-2 and CS-4 stages compared to CS-3 stage. 

The effect on mandibular incisors is variable in different studies. Lund and Sandler^35^ found a statistically significant increase in mandibular incisor inclination, while Illing et al^20^ found no significant change. In this study, a significant increase in mandibular incisor inclination was observed despite mandibular incisor capping into an appliance, which was found to be statistically significant amongst all cervical stages when compared to controls. Proclination of labial segment contributes to overjet reduction by limiting the potential for further growth. In addition, proclination of mandibular incisors increase the tendency towards relapse and, therefore, must be corrected during the second phase of orthodontic treatment with interdental stripping or extractions.^36^

### Soft tissue changes

#### Upper and lower lip position

Quintão et al^5^ found a significant change in upper lip position due to maxillary incisor retroclination after functional appliance treatment. In contrast, Morris et al,^17^ in their study, demonstrated no significant change in the sagittal position of upper lip despite large reductions in overjet. In our study, upper lip became significantly less projected in the treatment group when compared to the controls. Baysal and Uysal^3^ found greater advancement of the lower lip, lower lip sulcus and soft tissue pogonion in the Twin Block group. In contrast, Quintão et al,^5^ in their study, did not find any significant changes in any of the lower lip variables. In our study, lower lip changes were observed only in the CS-2 group. However, the E-line, as a reference plane to quantify actual changes in lips, is not very reliable because of the simultaneous growth of the soft tissue chin and pronasale that may give a false impression of the actual lip position. 

#### Nasolabial angle

Quintão et al,^5^ in their study, did not find any statistically significant change in the nasolabial angle after treatment with the Twin Block appliance. In contrast, Varlik et al^11^ found significant increase in nasolabial angle in the Twin Block group. Likewise, in our study, we found significant increase in the nasolabial angle, which may be the result of the change in upper lip position. On stratification of sample into different cervical stages, this increase was significant at the CS-4 stage when compared to controls.

#### Z-angle

Varlik et al,^11^ in their study, found a significant increase in Z-angle in patients treated with the Twin Block appliance due to forward movement of soft tissue chin. Our results are similar to their study. However, on stratification of sample into different cervical stages, this increase was significant only at the CS-3 stage when compared to controls.

#### H-angle

Holdaway^38^ related H-angle decreases as the facial convexity decreases. Baysal and Uysal,^3^ in their study, found a significant reduction in this angle after Twin Block appliance treatment, which showed improvement in facial convexity. In our study, we also found significant reduction in this angle at the CS-2 and CS-3 stages, with an overall improvement of facial profile. The possible explanation for this reduction in H-angle is the combination of upper lip retraction and forward movement of the soft tissue pogonion.

## CONCLUSIONS

" The Twin Block appliance reduces overjet in Class II, Division 1 malocclusion by means of favorable skeletal changes in bony bases and dentoalveolar compensations.

" Overlying soft tissues change along with underlying hard tissues, which improves overall facial esthetics. 

" Mandibular growth changes were significant amongst all cervical stages. However, they are more pronounced when appliance is placed during the CS-3 stage, as compared to CS-2 and CS-4 stages. Any attempt to change the growth is best achieved at the peak of pubertal growth; therefore, it is better to wait for CS-3 to achieve maximum skeletal effects as well as to reduce overall treatment duration.

" Dentoalveolar changes were also minimal during treatment in CS-3 stage, as compared to CS-2 and CS-4 stages.
